# The Impact of Early Epidural Analgesia on the Course of Labor and Delivery

**DOI:** 10.3390/medicina61040750

**Published:** 2025-04-18

**Authors:** Atene Simanauskaite, Gabriele Kavaliauskaite, Justina Kacerauskiene, Vilda Vilimiene

**Affiliations:** Faculty of Medicine, Medical Academy, Lithuanian University of Health Sciences, 44307 Kaunas, Lithuania; gabriele.kavaliauskaite@stud.lsmu.lt (G.K.); justina.kacerauskiene@lsmu.lt (J.K.); vilda.baliuliene@lsmu.lt (V.V.)

**Keywords:** early epidural analgesia, cervical dilation, nullipara

## Abstract

*Background and Objectives*: This study aimed to assess the impact of early epidural analgesia (EA) on the progression of labor and delivery outcomes among nulliparous women. *Materials and Methods*: A retrospective analysis was conducted utilizing data from the Birth Registry of the Department of Obstetrics and Gynecology at LUHS. The dataset encompassed women who underwent childbirth between 1 January 2021 and 31 December 2021 and who received EA for labor pain management. A total of 89 women with low-risk deliveries and EA were included in the study. The cohort was divided into two groups: Group I—parturients who underwent early EA with cervical dilatation ≤3 cm—and Group II—parturients who underwent EA with cervical dilatation >3 cm but <7 cm. The results were processed using IBM SPSS. *Results*: Group I consisted of 25 (28.1%) women and Group II consisted of 64 (71.9%). The prevalence of obesity was higher in Group II (*p* = 0.021). Bishop score was statistically elevated in Group II (*p* = 0.018). Upon hospital admission, Group II exhibited greater cervical dilation (*p* = 0.001). The rate of cervical dilation was higher in Group II at 1.54 cm/h (*p* = 0.033). Episiotomy was more frequently performed in Group II (*p* = 0.014). The average durations of the first stage of labor (*p* = 0.045), the second stage of labor (*p* = 0.033), and the overall labor (*p* = 0.023) were prolonged in Group I. *Conclusions*: The cervical dilation up to 10 cm occurs at a swifter pace when EA is administered following cervical dilation exceeding 3 cm. Notable associations were observed between EA and the incidence of episiotomy as well as the duration of labor stages. Early EA exhibited no impact on neonatal outcomes.

## 1. Introduction

Childbirth pain is widely recognized as one of the most intense forms of pain encountered by women. The perception of pain during childbirth varies individually, shaped by each woman’s unique experience and subjective interpretation [1]. A mere 10–15% of laboring women report minimal or mild pain, while approximately 35–40% contend with moderate pain, and a further 30–35% endure intense pain. In addition, 15–20% describe the pain as overwhelmingly intense and unbearable [2]. Severe labor pain can lead to hypertension, tachycardia, hyperventilation, and heart rhythm disorders, thus having a negative impact on the condition of the mother and the fetus, as well as on the course of labor. Consequently, approximately 50% of women in labor, who undergo severe or unbearable pain, necessitate pain relief measures [2,3,4].

Epidural analgesia (EA), as endorsed by the World Health Organization (WHO), stands as the gold standard for effective labor pain management [4,5]. Notably, in the United States of America (USA), over 60% of laboring women opt for regional analgesia as a means of alleviating labor-related pain [5]. Investigative surveys conducted in Poland in 2018 and Portugal in 2021 unveiled that mothers who received epidural pain relief during childbirth reported more positive birth experiences [6,7].

EA serves to alleviate the perception of pain, although it does not completely abolish sensory awareness [8]. Moreover, EA contributes to the reduction in stress hormone levels in both maternal and fetal contexts [9].

The timing for initiating epidural labor pain relief remains a topic of extensive discourse within the literature. A study by Chinese researchers advocated for EA as a viable option for all women upon request, irrespective of cervical dilation [10]. Nevertheless, a comprehensive literature review in 2018 exposed that this analgesic approach correlated with prolonged labor durations, an increased likelihood of labor augmentation, and heightened rates of instrumental deliveries [11,12,13]. While concerns persist regarding the potential interference of early EA with cervical cerclage and labor progression complexities [14], it is noteworthy that for primiparous women, EA can extend both the initial and subsequent labor stages [15]. Conversely, research from Israel implies that this method of analgesia contributes to a positive birthing experience and exerts minimal impact on the incidence of obstetric complications [12].

The objective of this study was to assess the impact of early epidural analgesia on the trajectory of labor and delivery outcomes among nulliparous women.

## 2. Materials and Methods

A retrospective study was conducted at the Obstetrics and Gynaecology Clinic of the Hospital of the Lithuanian University of Health Sciences, Kaunas Clinics, from 1st January 2021 to 31st December 2021. The study encompassed nulliparous women with low-risk pregnancies who received epidural analgesia (EA) for labor pain relief. The criteria for categorizing a pregnancy as low-risk were in accordance with the Obstetric Methodology “Antenatal Care” outlined by the Ministry of Health of the Republic of Lithuania. Such criteria considered the woman’s age to be between 18 and 40 years, the fetus to be mature, the pregnancy to be singleton, the fetus to exhibit cephalic presentation, and the labor to be spontaneous [16]. Data were meticulously extracted from birth registers, antenatal cards, and birth histories. The study was ethically sanctioned by the Bioethics Centre under the reference number BEC-MF-409.

Initially, a total of 96 women with low-risk deliveries and EA were identified within the designated timeframe. Subsequent data analysis revealed that 7 of these women exhibited an elevated risk of delivery, prompting their exclusion from the study. The remaining subjects were categorized into two distinct groups: Group I included parturients who underwent EA with cervical dilation ≤ 3 cm, while Group II included parturients who received EA with cervical dilation > 3 cm but <7 cm. Group I consisted of 25 (28.1%) women, and Group II consisted of 64 (71.9%).

Relevant obstetrical history data, including gestational age and unfavorable obstetrical history (such as preterm delivery and a prior miscarriage), were compiled and subjected to evaluation. Maternal height and weight were measured to derive the body mass index (BMI) (kg/m^2^). Pregnancy-associated weight gain was computed by quantifying the disparity between pre-pregnancy or initial antenatal visit weight (recorded in the antenatal record) and weight prior to delivery (recorded in the birth or last antenatal visit record).

The assessment of the cervix was executed in accordance with the modified Bishop score, encompassing the evaluation of cervical length, dilation, consistency, and position upon admission to the hospital and during EA administration. For all instances, a low concentration of bupivacaine (0.1%) was employed for analgesia. The interval for cervical ripening post-EA and the duration for complete cervical dilation were calculated.

Parameters such as the frequency of labor augmentation using oxytocin, the progress of labor, and labor outcomes (vaginal delivery, instrumental delivery using vacuum extraction, or emergency cesarean section) were meticulously examined. The duration of the initial and second labor phases, measured in hours, was recorded. The first labor phase commenced with the onset of contractions, leading to cervical dilation, and concluded upon reaching full 10 cm dilation. The second labor phase encompassed the period from complete cervical opening to fetal birth. Data concerning neonatal status post-birth, such as Apgar scores at 1 min and 5 min, along with newborn height and weight, were documented.

The data were collected and structured in Microsoft Office Excel and processed and analyzed using the statistical data analysis package IBM SPSS Statistics 23. The statistical relationship between two random samples of qualitative data was assessed using the chi-square (χ^2^) test, and Student’s *t*-tests were used to compare quantitative measurements. Results were considered statistically significant at *p* < 0.05. Percentages, frequencies, means, and standard deviations of continuous variables were calculated.

## 3. Results

There were 89 women included in the study: Group I comprised 25 women (28.1%) and Group II comprised 64 women (71.9%).

The comparison of maternal age in both groups is presented in Table 1. Pregestational weight exhibited a statistically significant elevation in Group II (*p* = 0.021). Additionally, the prevalence of obesity was notably higher within Group II (*p* = 0.007). Unfavorable obstetrical history was more often diagnosed in Group II (*p* = 0.047).

Characteristics of the cervix during admission to the hospital are presented in Table 2. Cervical alterations throughout labor were evaluated using the Bishop score. It was observed that women in Group II had a higher Bishop score compared to Group I, with a statistically significant difference (*p* = 0.018). The cervical length was significantly shorter, and the cervix was more dilated in Group II women during their admission to the hospital (*p* = 0.028 and *p* = 0.001, respectively).

Table 3 provides a comprehensive overview of cervical characteristics and their association with early epidural analgesia (EA). The mean cervical dilation prior to the administration of EA stood at 2.76 cm for participants in Group I, contrasting with 5.59 cm for those in Group II. Notably, following the initiation of EA, cervical dilation in Group I proceeded at a rate of 1.29 cm per hour, while Group II displayed a slightly swifter rate of 1.54 cm per hour.

Birth-related data are presented in Table 4. Episiotomy was significantly more often found in Group II (*p* = 0.014). The average duration of the first stage of labor (*p* = 0.045), the second stage of labor (*p* = 0.033), and total labor (*p* = 0.023) was longer in Group I. APGAR scores after 1 and 5 min after the birth were evaluated; there was no statistically significant difference.

The data related to newborns are shown in Table 5.

## 4. Discussion

The present study has effectively elucidated the impact of early epidural analgesia (EA) on cervical dilation among nulliparous women. When EA was administered with cervical dilation below 3 cm, a discernible extension in the average labor duration was observed. Furthermore, the initial and subsequent stages of labor exhibited prolonged durations in comparison to instances wherein epidural analgesia was administered during cervical dilation, ranging from 3 to 7 cm. Patient-related data have no difference between the study groups. Obesity was the only aspect that had a significant difference. In our study, obese patients used EA when cervical opening was >3 cm. Patient-specific data, for the most part, remained consistent across the study groups, with obesity emerging as the sole parameter that demonstrated notable significance. Specifically, our investigation highlighted that obese patients availed themselves of EA at a point when cervical dilation had exceeded 3 cm. This observation aligns with Tuija Hautakangas’s, which expounds on cervical dilation patterns in obese pregnant women. The relevant literature further suggests that obese women tend to experience contractions that are equally robust or even more intense than those of normal-weight women, yet they are at an increased risk of not attaining the active phase of labor [17].

In our study, the administration of EA was implemented for a significant proportion of nulliparous women once their cervical dilation had exceeded 3 cm. Correspondingly, analogous findings were elucidated in a cross-sectional study conducted by G.R. Abhirami. In this investigation, the administration of EA took place during the active phase of labor, with a notable statistic indicating that 70.2% of mothers underwent EA intervention when their cervical dilation had progressed to 4 cm [18].

Our study also highlighted a predilection among obese women to opt for EA. This inclination aligns with findings from a distinct study conducted by other researchers, wherein a comparative analysis of labor progression between obese and non-obese women revealed a statistically significant disparity in uterine tone and contractions across the two groups [19]. Moreover, the investigation indicated that obese women were more prone to receive oxytocin for labor augmentation in comparison to their normal-weight counterparts [20]. Notably, the utilization of oxytocin could potentially exert an influence on the subsequent inclination towards requesting EA during labor. This connection is attributed to oxytocin’s association with the inherent initiation of labor and its physiological role in mitigating fear and pain associated with the labor process [21].

In our study, the first and second stages of labor were significantly longer in Group I. The first stage of labor in Group I lasted about 61 min longer than in Group II, while the second stage of labor was about 43 min longer. Moreover, cervical dilation sped up to 10 cm after EA was faster in Group II. As in our results, similar data were found in other studies. These data showed that the early initiation of epidural analgesia (up to 3 cm cervix dilation) prolongs not only the second stage of labor [15] but also the total labor duration [14]. Comparable results were shown in a study in Ireland that the use of epidural anesthesia after a cervical dilation of more than 4 cm was significantly associated with a longer duration of the first and second stages of labor [13]. Moreover, the study conducted by other authors shows that the duration until full cervical dilation is prolonged for about 51 min in nulliparous women who undergo epidural anesthesia with cervical dilation ≤3 cm [14]. In our study, the cervix opening took about 106 min, which was twice as long as in the study conducted by other authors [13,14]. Today, it is considered that the optimal time to perform epidural anesthesia during labor should be when the cervical dilation is 6 cm or more, due to the fact that later epidural anesthesia administration is not associated with longer labor duration [19].

Within our study, bupivacaine emerged as the prevailing local anesthetic employed for epidural analgesia (EA). Traditionally, the practice entailed the utilization of a high concentration of local anesthetic, often ranging between 0.2% to 0.25%, to sustain the efficacy of EA. Over the course of time, a shift has occurred towards employing a combination of lower local anesthetic concentrations, typically falling below 0.1%, alongside lipophilic opioids. This strategic adjustment has yielded notable benefits, notably a reduction in adverse events like motor blockage and hypotension [20]. In alignment with this progression, our study consistently utilized a local anesthetic concentration of 1 mg/mL. A study undertaken by L. Halliday et al. contributes to the literature, emphasizing that the adoption of low or ultra-low concentrations of local anesthetic associates with an amplified likelihood of spontaneous vaginal delivery and a shortened duration of the second stage of labor [21].

The introduction of early EA has been associated with a decelerated pace of labor progression and an augmented likelihood of cesarean section (CS) [11]. However, the findings obtained from our study, along with outcomes from parallel investigations, do not reveal a substantial disparity in CS rates attributable to EA [12,22]. Notably, viewpoints echoed by other authors suggest that EA can be regarded as a risk factor for a heightened incidence of instrumental deliveries [23,24,25]. Furthermore, the research conducted by N. Srebnik et al. underscores that the EA-induced prolongation of the second labor stage translates to an increased risk of instrumental delivery [24]. Interestingly, our study did not yield a noteworthy contrast in the incidence of instrumental deliveries based on EA utilization.

Episiotomy procedures were observed to occur with greater frequency within group II. The Institute of Hygiene Health Information Centre, responsible for monitoring Lithuania’s population health and healthcare resources, has reported an escalating trend in the rate of episiotomy since the year 2020 [25]. Expanding on this matter, G. Baczek et al.’s investigation highlights that epidural analgesia contributes to alterations in the delivery mechanism, resulting in a more than fivefold increase in supplementary medical procedures like episiotomy and a more than twofold surge in perineal lacerations [26]. The underpinning mechanism fostering the correlation between EA and heightened episiotomy rates is presumed to be multifaceted, potentially involving factors such as the diminished urge to push due to the attenuation of the bearing-down reflex, curtailed uterine activity, and diminished sensation [19].

Another inquiry, encompassing a population of 23,272 low-risk women, discerned that EA during labor among low-risk pregnant women is linked with diminished Apgar scores in neonates [27]. Nevertheless, neither our study nor the works of other researchers have unveiled a significant association between low Apgar scores and the utilization of EA [12,28].

## 5. Conclusions

Cervical dilation up to 10 cm occurs at a swifter pace when early epidural analgesia (EA) is administered after cervical dilation surpasses 3 cm. Moreover, when EA is administered during cervical dilation less than 3 cm, it impacts the extension of the durations of both the first and second labor stages. Meanwhile, the correlation between EA and cesarean section (CS) or instrumental delivery did not exhibit statistical significance. Early EA exhibited no impact on neonatal outcomes.

## Figures and Tables

**Table 1:** Comparison of anthropometric data and obstetrical history of the patients.

	Group I	Group II	*p* Value
	*n* = 25	*n* = 64	
Patient’s age (years, SD)	30 (±1.2)	28 (±0.5)	0.202
Height, cm (SD)	168.2 (±6.2)	169.5 (±5.9)	0.219
Pregestational weight, kg (SD)	62.7 (±8.9)	74.1 (±17.2)	0.021
Pregestational BMI, kg/m^2^ (SD)	22.3 (±2.8)	22.8 (±3.9)	0.550
Obesity, *n* (%)	1 (4)	11 (17.2)	0.007
Weight gain during pregnancy, kg (SD)	15.3 (±4.8)	14.4 (±5.1)	0.440
Gestational age, weeks (SD)	39.0 (±1.1)	40.0 (±1.0)	0.125
Unfavorable obstetrical history, *n* (%)	1 (4)	8 (12.5)	0.047

*p* value < 0.05 was considered to indicate a statistically significant difference.

**Table 2:** Characteristics of the cervix when women were admitted to the hospital.

		Group I	Group II	*p* Value
		*n* = 25	*n* = 64	
Bishop score (SD)		4.7 (±1.6)	5.9 (±2.2)	0.018
Cervical length, cm (SD)		1.44 (±1.1)	0.98 (±0.8)	0.028
Cervical dilation, cm (SD)		1.82 (±0.9)	2.79 (±1.1)	0.001
Cervical consistency	Firm, *n* (%)	4 (16)	6 (9.4)	0.007
	Moderate, *n* (%)	10 (40)	32 (50)	0.195
	Soft, *n* (%)	11 (44)	26 (40.6)	0.613
Cervical position	Middle, *n* (%)	14 (56)	35 (54.7)	0.545
	Anterior, *n* (%)	1 (4)	4 (6.3)	0.325
	Posterior, *n* (%)	10 (40)	25 (39)	0.532

*p* value < 0.05 was considered to indicate a statistically significant difference.

**Table 3:** The comparison of cervical dilation and its relation to EA.

	Group I	Group II	*p* Value
	*n* = 25	*n* = 64	
Cervical dilation before EA, cm (SD)	2.76 (±0.5)	5.59 (±1.1)	0.001
Time of full cervical dilation after EA, min (SD)	379 (±249)	273 (±138)	0.018
Cervical dilation speed up to 10 cm after EA, cm/h (SD)	1.29 (±0.75)	1.54 (±1.1)	0.033

*p* value < 0.05 was considered to indicate a statistically significant difference.

**Table 4:** Birth-related data.

		Group I	Group II	*p* Value
		*n* = 25	*n* = 64	
Labor	Vaginal birth, *n* (%)	21 (84)	57 (89.1)	0.271
	Cesarean section (total number), *n* (%)	4 (4,0)	7 (10.9)	0.729
Oxytocin, *n* (%)		3 (12)	8 (12.5)	0.474
Episiotomy, *n* (%)		11 (44)	36 (56.3)	0.014
Instrumental labor, *n* (%)		1 (4)	1 (1.6)	0.719
Total labor duration, h (SD)		975 min (±376 min)	854 min (±259 min)	0.023
Stages of labor	The first stage of labor, h (SD)	887 min (±460 min)	826 min (±293 min)	0.045
	The second stage of labor, h (SD)	107 min (±85 min)	70 min (±76 min)	0.033

*p* value < 0.05 was considered to indicate a statistically significant difference.

**Table 5:** The comparison of newborns.

		Group I	Group II	*p* Value
		*n* = 25	*n* = 64	
Height of newborn, cm (SD)		52.4 (±2.2)	52 (±1.8)	0.469
Weight of newborn, g (SD)		3456.4 (±380.8)	3471.3 (±356.5)	0.425
APGAR	Apgar after 1 min (SD)	9 (±0.9)	9 (±1.1)	0.711
	Apgar after 5 min (SD)	10 (±0.7)	10 (±0.6)	0.666

## Data Availability

The original contributions presented in this study are included in the article. Further inquiries can be directed to the corresponding author.
